# Development and usability testing of a preliminary web-based application for the clinical implementation of blood flow restriction: a mixed methods pilot study

**DOI:** 10.3389/fphys.2025.1631562

**Published:** 2025-07-15

**Authors:** Isaac J. Wedig, Erich J. Petushek, John J. Durocher, John McDaniel, Steven J. Elmer

**Affiliations:** ^1^ School of Health and Human Performance, Northern Michigan University, Marquette, MI, United States; ^2^ Department of Kinesiology and Integrative Physiology, Michigan Technological University, Houghton, MI, United States; ^3^ Health Research Institute, Michigan Technological University, Houghton, MI, United States; ^4^ Department of Psychology and Human Factors, Michigan Technological University, Houghton, MI, United States; ^5^ Department of Biological Sciences and Integrative Physiology and Health Sciences Center, Purdue University Northwest, Hammond, IN, United States; ^6^ Department of Exercise Physiology, Kent State University, Kent, OH, United States; ^7^ Doctor of Physical Therapy Program, St. Catherine University, Saint Paul, MN, United States

**Keywords:** physical therapy, occlusion training, limb occlusion pressure, arterial occlusion pressure, rehabilitation, decision support tool

## Abstract

**Introduction:**

Exercise with blood flow restriction (BFR) has gained popularity for use with a wide range of healthy and clinical populations. However, several factors including medical screening, selection of equipment, and determination of cuff pressure still pose barriers for implementation. Accordingly, this study aimed to develop and test a web-based application to guide practitioners in using BFR safely and effectively.

**Methods:**

First, we developed an application to assist with medical screening, selection of appropriate equipment, and determination of cuff pressures. Subsequently, we conducted preliminary usability testing of the application using a mixed methods approach. Licensed physical therapists (n = 5) with no prior experience with BFR used the application to implement BFR exercise in hypothetical patient scenarios. Afterward, perceived usability was assessed using the System Usability Scale (SUS) and semi-structured interviews analyzed through thematic analysis.

**Results:**

All task scenarios were successfully completed in an average time of 2.3 ± 1.2 min. A total of 11 errors occurred, including minor navigation issues (4), data input problems (2), and difficulty interpreting recommendations (5). The composite SUS score was 94 ± 5, ranking highly compared to industry standards. Interviews revealed that the application was efficient, boosted confidence in using BFR, and increased the perceived likelihood of incorporating BFR into clinical practice.

**Discussion:**

These findings suggest that the web-based application has potential to serve as a valuable tool for overcoming barriers to BFR use, enhancing accessibility, and improving the safety and effectiveness of BFR implementation in clinical settings.

## Introduction

Exercise with blood flow restriction (BFR) offers a unique approach for increasing muscle size and strength (Lixandrão et al., 2018; Slysz et al., 2016; Loenneke et al., 2012b; Grønfeldt et al., 2020), aerobic capacity (Formiga et al., 2020; Bennett and Slattery, 2019), and physical function (Clarkson et al., 2019) in healthy adults. Emerging evidence indicates that this modality may be an effective exercise option for a broad range of clinical populations including those individuals living with hypertension (Wong et al., 2018), cardiovascular disease (Kambič et al., 2019; Ogawa et al., 2021; Madarame et al., 2013; Ishizaka et al., 2019; Nakajima et al., 2010; Fukuda et al., 2013), diabetes (Malekyian Fini et al., 2021; Fini et al., 2021), renal dysfunction (Corrêa et al., 2021b; Corrêa et al., 2021a), and musculoskeletal conditions (Hughes et al., 2017; Kong et al., 2022; Pitsillides et al., 2021; Lu et al., 2020). Accordingly, exercise with BFR is now endorsed by the American Physical Therapy Association (2018) and used in rehabilitation (Patterson and Brandner, 2018; Colapietro et al., 2022; Mills et al., 2021; Castle et al., 2023; Scott et al., 2024; Cuffe et al., 2022).

Despite its growing use, implementation of exercise with BFR presents challenges for some practitioners (LaPrade et al., 2021). Most notably, methods used to implement exercise with BFR vary considerably (Fahs et al., 2012; Rolnick et al., 2023; Hughes et al., 2025; Freitas et al., 2021) with different equipment (i.e., pneumatic cuffs and/or elastic wraps of varying width, shape, and material), procedures for determining cuff pressure [i.e., arbitrarily selected or based on perceived tightness, systolic blood pressure, limb circumference, or arterial occlusion pressure (AOP)], and a wide range of applied cuff pressures (e.g., 100–240 mmHg or 40%–80% AOP). With this in mind, insufficient training and education, lack of access to equipment, and safety concerns often pose barriers to BFR implementation (Scott et al., 2024; Mills et al., 2021; Cuffe et al., 2022; Rolnick et al., 2021). Rolnick et al. (2021) highlighted three specific methodological obstacles including the conducting of systematic medical screening for safe BFR inclusion, selection of appropriate training equipment for performing BFR, and determining cuff pressures to utilize during exercise. Circumventing these barriers is critical to improving access to BFR and helping to ensure that safe and effective practices are utilized. Currently, general recommendations for performing BFR are available (Patterson et al., 2019; Cognetti et al., 2022), however, to the best of our knowledge there are no standardized methods published and/or comprehensive guides available for practitioners to follow. Specifically, an evidence-based tool that guides practitioners through the process of medically screening candidates for BFR inclusion, selecting appropriate training equipment, and setting proper cuff pressures is needed to bridge the gap between research and practice and enhance use of BFR in rehabilitation.

With the emerging use of smart devices such as mobile phones, tablets, and laptop computers in healthcare, there has been increased development and use of digital medical software applications (Boulos et al., 2011; Franko and Tirrell, 2012). Some evidence (Ventola, 2014) indicates that mobile and web-based applications increase productivity, enhance access to point-of-care tools, and improve clinical decision making and patient outcomes. Numerous mobile and web-based applications (Boland et al., 2016; Wellmon et al., 2016; Hansen et al., 2017; Peart et al., 2019; Muntaner-Mas et al., 2019; Deutsch et al., 2015; Srikesavan et al., 2017; Rhodes et al., 2019) have been developed to assist physical therapists, in particular, in clinical decision making. Furthermore, Alsobhi et al. (2022) reported that physical therapists’ attitudes regarding the use of applications in clinical practice were positive, with the majority agreeing that they can be used as an assistive technology, used to enhance education, and facilitate patient care. Thus, a mobile and/or web-based application could provide physical therapists with a decision support tool to aid in the implementation of exercise with BFR.

An important factor to consider when developing digital applications is usability. Specifically, usability refers to the effectiveness, efficiency, and satisfaction with which a system can be utilized to complete a task in an intended group of users (Bevana et al., 1991). Evaluating usability is a critical step in the user centered design process of interactive technological systems (Organisation, 1999; International Organization for Standardization, 2018) and helps to identify design flaws and improve adoption and effectiveness of the tool in an end user. To date, numerous applications developed to assist physical therapists in education and clinical decision making have been usability tested (Baschung Pfister et al., 2020; Hartstein et al., 2022; Åström and Sahlin, 2022; Deutsch et al., 2015; Srikesavan et al., 2017; Nast et al., 2024; Rhodes et al., 2019). However, only a small number of health related applications available for commercial use have published usability evaluations (Maramba et al., 2019). For successful development and clinical adoption of an application designed to aid in BFR implementation, it is critical that usability testing be conducted in the intended user and published to ensure that the application is effective, efficient, and satisfactory for use by physical therapists.

The purpose of this pilot study was to 1) describe the development of a preliminary web-based application to aid in the implementation of exercise with BFR and 2) conduct initial usability testing of the web-based application in a small sample of physical therapists with no prior experience using exercise with BFR. Importantly, an iterative process of usability testing performed early and frequently can provide continuous feedback throughout the design process (Genov, 2005). Therefore, developing a preliminary application and performing early usability testing in the target audience would help to identify potential issues in the initial design and lay the groundwork for a future version that can be validated for clinical use.

## Methods

### Study overview

**FIGURE 1:**
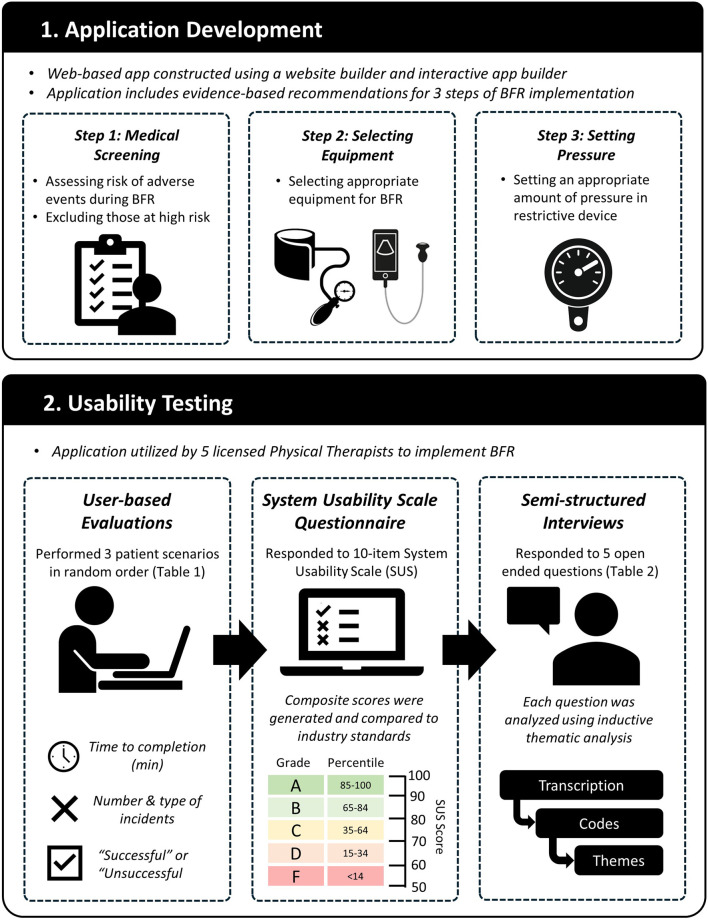
Study overview including application development and usability testing.

A web-based application was developed to aid in evidence-based implementation of exercise with BFR. We utilized a mixed methods approach to evaluate the usability of the developed web-based application with physical therapists. Participants attended one virtual meeting held on the Zoom platform (Zoom Cloud Meetings, version 5.12.9, San Jose, CA, United States). First, they were introduced to the web-based application and given a brief description of its purpose. Next, a user-based evaluation was conducted in which participants were given several scenarios and were asked to use the web-based application to complete a series of tasks. Following the user-based evaluation, participants ratings of perceived usability of the web-based application were evaluated using the System Usability Scale (Brooke, 1996). Lastly, semi-structured interviews were conducted consisting of a series of open-ended questions to elicit additional feedback. Interviews were qualitatively analyzed to identify themes across participant responses to each question. An overview of the study is displayed in Figure 1.

### Application development

We created a web-based application using a commercially available website builder (Squarespace, New York City, NY, United States). Several interactive web applications were constructed using Shiny (Shiny: https://www.shinyapps.io/). These applications were published to the internet using shinyapps. io (Posit Software) and were embedded into pages of the website. Collectively, the web-based application was developed to guide physical therapists through three primary steps of implementing exercise with BFR that have been previously identified as barriers. Specifically, steps included Step 1: Medical Screening, Step 2: Selecting Equipment, and Step 3: Determining Cuff Pressure. An overview of the purpose and evidence-based rationale used to develop the functions and procedures included in each step is described below.

#### Step 1: medical screening

The relative safety of performing exercise with BFR is an important concern (Loenneke et al., 2011; Brandner et al., 2018; Cristina-Oliveira et al., 2020; Kacin et al., 2015). Several potential contraindications and risk factors have been identified that may increase risk for adverse events. Accordingly, reviewing an individual’s lifestyle and medical history is important in stratifying risk and excluding those individuals from BFR participation in which risk may be heightened. The purpose of this step was to help physical therapists conduct medical screening of potential candidates and stratify the risk of adverse events. Several authors (Rolnick et al., 2021; Kacin et al., 2015; Nakajima et al., 2011; Nascimento et al., 2022) have developed tools to stratify risk and screen individuals for BFR inclusion. Existing screening tools were collected and used to develop a preliminary interactive medical screening application using Shiny.

#### Step 2: selecting equipment

Three main types of equipment have been used to implement exercise with BFR. These include automated pneumatic cuff systems (Jacobs et al., 2023; Hughes and McEwen, 2021), manual pneumatic cuffs (Loenneke et al., 2012a), and elastic wraps (Loenneke and Pujol, 2009). The type of equipment utilized can impact the physiological and perceptual responses to exercise with BFR and may play a role in modulating risk of adverse events. Specifically, pneumatic cuff systems (i.e., automated and manual) allow for more precise and standardized selection of external pressure applied to limbs compared to elastic wraps (Bell et al., 2020). Furthermore, some automated pneumatic cuff systems supply constant applied pressures (i.e., autoregulated) during exercise which attenuates perceptual and hemodynamic responses (Hughes et al., 2018) and reduces incidence of adverse events (Jacobs et al., 2023). Accordingly, the purpose of this step was to recommend appropriate equipment for implementing exercise with BFR based on results of the medical screening conducted in Step 1. The user would then be free to select from recommended equipment types based on accessibility. Given that practitioners may not have knowledge of different BFR equipment types, we aimed to provide resources that would help to describe the equipment and direct practitioners to commercially available products that to our knowledge have been validated.

#### Step 3: determining cuff pressure

The amount of external pressure applied to the limb during exercise with BFR is an important methodological consideration for safety and effectiveness. When utilizing pneumatic cuff systems, it is recommended (Patterson et al., 2019; McEwen et al., 2019) that pressures during exercise with BFR be selected based on arterial occlusion pressure (AOP) which is the minimum amount of pressure required to occlude arterial blood flow to the limb. Thus, for use of pneumatic cuff systems, the web-based application was designed to 1) help users determine AOP, and 2) recommend exercising cuff pressures based on that value.

Several methods for determining AOP are available. Automated pneumatic cuff systems have built in sensors for determining AOP (Hughes and McEwen, 2021), whereas manual pneumatic cuffs require direct measurement of AOP using pulse oximetry (Brekke et al., 2020; Lima-Soares et al., 2022), handheld, or ultrasound Doppler (Loenneke et al., 2012a). For practitioners that may not have access to this equipment, an alternative approach is estimating AOP based on anthropometric, blood pressure, and sociodemographic variables. Our laboratory and several authors (Cirilo-Sousa et al., 2019; Loenneke et al., 2015; Loenneke et al., 2012a; Sieljacks et al., 2018; Jessee et al., 2016; Wedig et al., 2024) have developed prediction equations to estimate AOP for a variety of manual pneumatic cuffs of different width. The web-based application was designed to guide users through each of the different methods of determining and/or estimating AOP based on equipment availability. To aid in AOP estimation, we developed an interactive application using Shiny that integrates prediction equations for 5, 11, 13, and 18 cm wide cuffs. Equations from Loenneke et al. (2015) were used for estimating upper and lower-body AOP with a 5 cm wide cuff. Both published (Wedig et al., 2024) and unpublished prediction equations developed by our laboratory were used for estimating lower-body AOP for 18 cm wide and 11 and 13 cm wide cuffs, respectively.

Evidence (Patterson et al., 2019) indicates that pressures between 40% and 80% of AOP are effective in promoting muscular adaptations during exercise with BFR. However, lower pressures within this range attenuate acute cardiovascular and perceptual responses (Spitz et al., 2022; Jessee et al., 2017; Mattocks et al., 2017) such as blood pressure, pain, and discomfort during exercise with BFR and represent safer options for those with increased risk of adverse events. Thus, the web-based application was designed to provide specific pressure recommendations relative to AOP that are based on the results of medical screening obtained in Step 1. We developed an interactive application using Shiny that provides exercising cuff pressure recommendations based on AOP values input by the user.

Determining exercising pressure relative to AOP is not possible when utilizing elastic wraps. Several approaches (Aniceto and da Silva Leandro, 2022) have been suggested for applying an appropriate amount of external pressure when utilizing this type of equipment to implement exercise with BFR. Limb circumference has been identified as the primary determinant of AOP when utilizing pneumatic cuffs (Cirilo-Sousa et al., 2019; Loenneke et al., 2015; Loenneke et al., 2012a). Therefore, authors (Aniceto and da Silva Leandro, 2022) have suggested that when utilizing elastic wraps for BFR, approaches to quantifying tightness of the wraps that are based on the circumference of the limb offer the most standardized method. Accordingly, the web-based application aimed to provide instructions on how to utilize these approaches for users choosing to implement exercise with BFR using elastic wraps.

### Participants

Five licensed Physical Therapists (30 ± 4 years, male = 2, female = 3) were recruited to participate in the study. A list of known physical therapists was created and participants from this list were recruited through email and/or phone calls. Participants had 5 ± 5 years of experience working in outpatient rehabilitation settings. Participants had heard of BFR previously, however, none had any prior experience implementing exercise with BFR in clinical practice. Usability trials (Nielsen and Landauer, 1993; Virzi, 1992; Virzi, 1990) have demonstrated that a sample of five participants can identify 80% of usability issues and that further participants become less likely to identify new issues. Accordingly, we utilized a convenience sample of five participants for our initial round of usability testing. Participants were informed of the purpose of the study and gave verbal consent. This study was approved by the Institutional Review Board at Michigan Technological University.

### Usability testing

#### User-based evaluation

Participants were given three scenarios, each consisting of a hypothetical patient, a reason for physical therapy treatment, and a specific goal for using BFR (Table 1). For each scenario participants were asked to use the web-based application to complete three tasks; 1) determine whether it was safe for the patient to engage in exercise with BFR, 2) select equipment for performing BFR based on what they were most likely to have access to, and 3) determine how much pressure to apply with the selected equipment during exercise. Patient scenarios were given to participants in a randomized order. Prior to beginning the task scenarios, participants were asked to share their computer screen and display the application webpage. While working through the assigned tasks, participants were instructed to use the “think aloud” (Jaspers et al., 2004) method by verbally walking through their thought process. The time taken to complete all three tasks, the number of incidents encountered, and type of incidents were recorded during each scenario. To explore which types of equipment participants had access to for implementing BFR, the type of equipment selected during task 2 and the method of determining cuff pressure during task 3 were recorded for each scenario. Each scenario was categorized as “Successful” or “Unsuccessful” based on whether an appropriate pressure was selected for the hypothetical patient in task 3.

**TABLE 1:** Scenarios given to each participant during user-based evaluation.

Information	Scenario 1	Scenario 2	Scenario 3
Patient	62-year old Female	30-year old Male	50-year old Male
Cause for treatment	Osteoarthritis	Patellofemoral pain	Post ACL reconstruction
Goal of BFR	Increase lower-body strength	Maintain strength lower-body strength	Regain lower-body strength
Characteristics	BMI: 31 BP: 135/90 mmHg	BMI: 24 BP: 125/82 mmHg TC: 60 cm	BMI: 20 BP: 118/78 mmHg TC: 52 cm
Health history	Diabetes Varicose veins in legs	NA	Surgery in last 4-weeks

#### System usability scale

Perceived usability of the application was evaluated using the System Usability Scale (SUS) (Brooke, 1996). A SUS questionnaire was administered to participants using Google Forms. The SUS is a 10-item scale that examines the perceived usability of a technological tool. Responses are assessed on a Likert scale ranging from 1 (Strongly Disagree) to 5 (Strongly Agree). Responses on each item can be evaluated individually to determine specific usability issues and/or used to generate a composite SUS score between 0 and 100, with higher scores indicating higher perceptions of usability (Lewis and Sauro, 2018). The SUS has been widely utilized which allows for relative comparison of SUS scores based on normative data. Importantly, the SUS is a valid (Bangor et al., 2008; Peres et al., 2013; Lewis et al., 2015) tool when assessing the usability of mobile applications and websites.

#### Semi-structured interviews

Participants were asked to respond to a set of 5 open-ended questions (Table 2). Questions were designed to collect feedback pertaining to the usability of the web-based application. Interviews lasted between 10 and 30 min (17 ± 6 min). Audio recordings of interviews were transcribed for analysis.

**TABLE 2:** Semi-structured interview questions.

Questions
1. Is a there a specific reason why you have not utilized exercise with blood flow restriction in your clinical practice?
2. What are some perceived barriers to implementing exercise with blood flow restriction in your clinical practice?
3. What aspects of this web-based application did you find helpful in implementing exercise with blood flow restriction?
4. How could this web-based application be improved to help you implement BFR more confidently?
5. If this application was available, how do you think that it would change the use of blood flow restriction in clinical practice?

**TABLE 3:** Medical screening and risk stratification Point system.

Absolute risks	
Points	Risk factor
5	Family history of clotting disorders
5	History of deep vein thrombosis or pulmonary emboli
5	History of hemorrhagic shock
5	Systolic blood pressure ≥ 140 mmHg

Relative risks	
Points	Risk factor
4	Pregnant
4	Diabetes
4	Systolic blood pressure 120–140 mmHg
4	History of injury to arteries or veins
4	History of surgery in past 4 weeks
4	Journey lasting more than 4 h or a flight in last 7 days
3	Atrial fibrillation, heart failure, or other cardiovascular disease
3	Varicose veins in the legs
3	Bruise easily
3	Prolonged immobility (lasting > 8 h) in last 7 days
2	Using oral contraceptives or hormone replacement
2	Hyperlipidemia
2	Malignant Cancer
2	People over the age of 60 years old
2	BMI > 30
1	Smoke or use nicotine products
1	People aged 40–58 years old
1	Women
1	25 < BMI < 30

Points were additive across all identified risk factors. Individuals accumulating ≥ 5 points were categorized as “High Risk,” 4 points as “Moderate Risk,” 3 points as “Low Risk,” and ≤ 2 points as “Very Low Risk.”

### Data analysis

Descriptive statistics were used to analyze time to task completion and the number of incidents while completing each task scenario. The type of incidents was qualitatively analyzed across all participants and placed into categories. For SUS responses, composite scores between 0 and 100 were calculated according to procedures described by Brooke (Brooke, 1996). Means and standard deviations were calculated for composite scores and for each individual response item. Composite SUS scores were interpreted relative to industry percentiles using a curved graded scale formulated by Lewis and Sauro (Lewis and Sauro, 2018). Individual item responses were interpreted by comparison to item benchmarks (Lewis and Sauro, 2018) established for SUS scores of 68 and 80. These item benchmarks represent mean Likert scale responses for each individual item that correspond to SUS composite scores at the 50th (SUS score 68) and 90th (SUS score 80) percentile of industry standards. Transcripts of semi-structured interviews were qualitatively analyzed using inductive thematic analysis as described by Braun and Clarke (Braun and Clarke, 2006). Six phases of analysis were utilized including: 1) familiarization with the data, 2) generating initial codes, 3) searching for themes, 4) reviewing themes, and 5) defining and naming themes, and 6) generating a report. Initial familiarization with the data was performed by IJW and consisted of re-reading interview transcripts while extracting meaning and patterns. Initial codes were developed by IJW using an inductive approach. Lastly, themes and subthemes were developed by establishing possible relationships between codes. Saturation in thematic analysis was reached within our sample and was defined as the point when no new codes were identified in two consecutive interviews. Importantly, it is not rare to achieve saturation with a small sample size when samples are highly homogenous (Hennink and Kaiser, 2022).

## Results

### Application development

**FIGURE 2:**
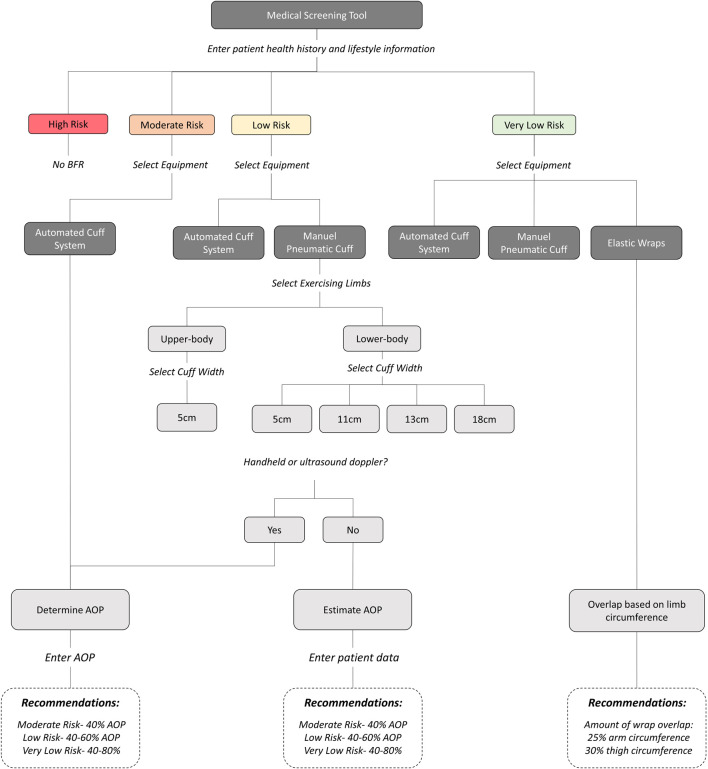
Workflow of the web-based application.

A web-based application called “BFR Exercise Trainer” was developed (https://tiger-bobcat-mjjk.squarespace.com/). A detailed overview of the application workflow is displayed in Figure 2. A description of features and evidence-based recommendations provided within each step of the application are described below.

#### Step 1: medical screening

**FIGURE 3:**
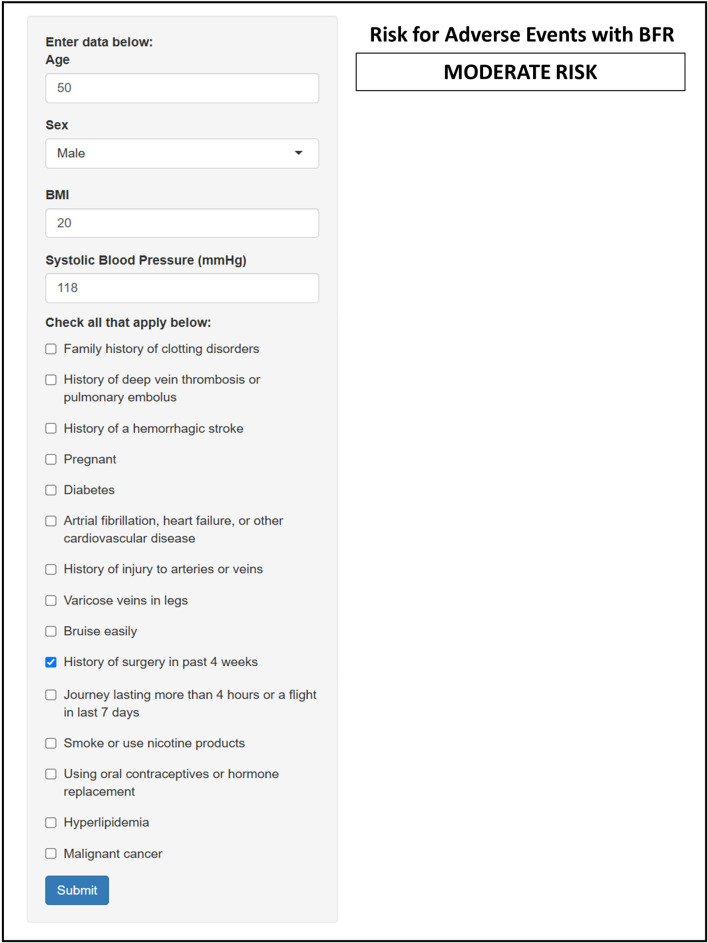
An image of medical screening within the web-based application.

We utilized screening tools previously suggested by Kacin et al. (2015) and Nakajima et al. (2011) to develop a modified risk stratification tool. These screening tools were selected because, to the best of our knowledge at the time of application development, they represented the most comprehensive yet practical options being both simple and easy to complete. Kacin et al. (2015) separated risk factors into “absolute” and “relative,” in which those with absolute risk factors are automatically excluded from exercise with BFR and those with relative risk factors are prompted to seek medical advice. Nakajima et al. (2011) proposed a point-based risk scoring system previously utilized by surgeons to assess risk of pulmonary embolism and deep-vein thrombosis. Risk factors are assigned a point value (1–5) based on the level of relative risk that they incur, and points associated with each risk factor are additive. Those individuals accumulating 5 or more risk points are excluded from performing exercise with BFR. We integrated the two screening tools together by using the risk point system described by Nakajima et al. (2011) and included any additional absolute and relative risks further described by Kacin et al. (2015). All absolute risks from Kacin et al. (2015) were each assigned a point value of 5 whereas the relative risks were assigned a point value of 1 to 4. All risk factors included in our hybrid screening tool and their associated point values are listed in Table 3. The hybrid medical screening tool was integrated into an interactive Shiny application and embedded on a medical screening page within the web-based application (Figure 3). The user enters a patient’s medical history and lifestyle information into the input field of the application and is provided with a risk classification based on the number of points accumulated from all identified factors. The accumulation of ≥ 5 points classify individuals as “High Risk” and the application suggests that individuals be excluded from BFR. An accumulation of 4 risk points is classified as “Moderate Risk” and users are prompted to seek medical clearance from a primary care provider before engaging in exercise with BFR. An accumulation of ≤ 3 risk points (3 = “Low”, ≤ 2 = “Very Low”) suggests that exercise with BFR is not an absolute contraindication and that it can be performed. Therefore, users selecting a risk classification of “Low” or “Very Low” are prompted to move on to Step 2.

#### Step 2: selecting technologies

**FIGURE 4:**
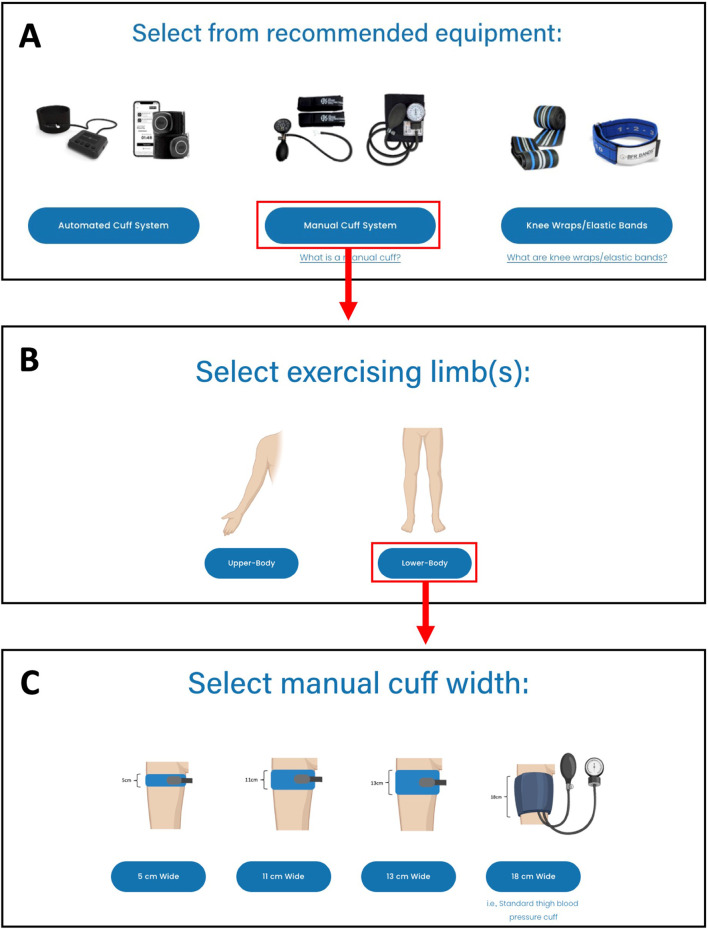
An image of Step 2: Selecting Equipment for performing exercise with BFR with the web-based application. Example shows the selection of a manual pneumatic cuff. **(A)** User selects from recommended equipment types, **(B)** User selects the limbs where BFR exercise will be performed, **(C)** User selects the cuff width that will be used.

Recommended equipment for performing exercise with BFR was provided to users based on the risk stratification resulting in Step 1. For patients with a “Moderate Risk”, the application provides users with the option to utilize automated cuff systems only. For patients with “Low Risk” the option of choosing either an automated cuff system or a manual pneumatic cuff was provided. Lastly, for patients with a “Very Low Risk”, users were given the option to choose from an automated cuff system, a manual cuff system, or elastic wraps. Selection of an automated cuff system or elastic wraps brought the user to the next step (Step 3). Selection of manual pneumatic cuffs prompted the user to select whether BFR will be performed in the lower- or upper-body. The user is then given the option to select from manual cuff widths commonly used for performing exercise with BFR in the selected limb (Upper-body: 5 cm wide, Lower-body: 5 cm, 11 cm, 13 cm, or 18 cm). After selecting a specific cuff width, the user is brought to Step 3. An illustration of Step 2 is provided in Figure 4. For each type of equipment recommended to users, a description of the equipment and links to commercially available products were provided.

#### Step 3: selecting restriction pressure

**FIGURE 5:**
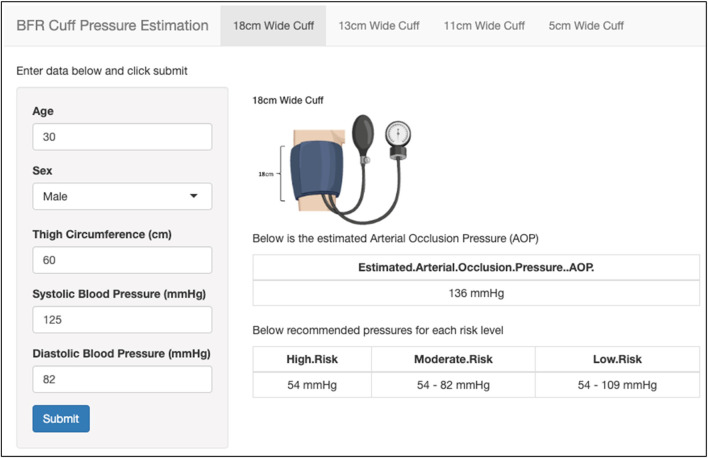
An image of estimating AOP within the web-based application. User selects the width of cuff (top), enters relevant predictor variables (left), and is provided with cuff pressure recommendations based on the estimated AOP (right).

Users that selected to utilize pneumatic cuff systems (i.e., automated and manual) were prompted to determine AOP. When manual cuff systems are selected, the user is asked if they have access to equipment for assessing AOP directly (i.e., handheld or ultrasound Doppler). If they select “Yes,”, they are brought to a page with instructions on how to measure AOP and provided links to video demonstrations for measuring AOP in both the upper- and lower-body. If they select “No,” they are brought to a webpage that helps them to estimate AOP using the Shiny application with integrated prediction equations (Wedig et al., 2024; Loenneke et al., 2015) (Figure 5). Within the application, the user selects the width of the manual cuff to be utilized and is provided with fields to input relevant predictor variables required (i.e., age, sex, limb circumference, systolic and diastolic blood pressure) for each respective prediction equation. Output from the application includes an estimated AOP for the selected cuff width.

**FIGURE 6:**
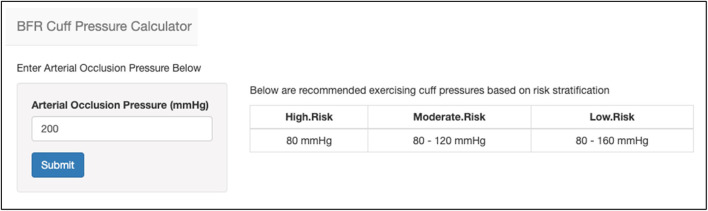
An image of the BFR cuff pressure calculator that provides recommended exercising cuff pressures during BFR based on AOP. The user provides an AOP value (left) and is provided with recommended pressures to utilize based on the risk stratification level obtained during medical screening (right).

After AOP is either measured directly or estimated, pressures to utilize during exercise are provided relative to that value. Specific exercising pressure recommendations were given based on a patient’s risk stratification obtained in Step 1. Pressures equivalent to 40% AOP are recommended for those with “Moderate Risk,” 40%–60% AOP for those with “Low Risk”, and 40%–80% AOP for those with “Very Low Risk.” For those using automated cuffs or measuring AOP directly, users are prompted to use a Shiny application to enter the AOP value. The application then provides output of specific pressure recommendations based on the risk stratification levels stated above (Figure 6). For those choosing to estimate AOP, pressure recommendations are provided within the Shiny application based on the estimated AOP value.

The option to utilize elastic wraps for performing exercise with BFR is only provided to those individuals with a “Very Low” risk classification obtained during medical screening. As the amount of pressure (i.e., mmHg) cannot be quantified for this type of equipment, the selection of elastic wraps provides the user with instructions on how tightly to apply the wraps during exercise. Specifically, users are provided with step-by-step directions for applying wraps based on the amount of overlap in the wrap relative to limb circumference as described by Aniceto and da Silva Leandro (2022) and Abe et al. (2019). Instructions are provided for applying the specific type of elastic wrap utilized by these authors (Harbinger Red-Line, Fairfield, CA, United States ; 7.6 cm width). For the upper limbs, users are instructed to measure the circumference of the upper arm and to apply the wrap so that it is stretched to a length corresponding to 25% of the resting arm circumference during each revolution around the limb. For the lower limbs, users are instructed to measure the circumference of the thigh and apply the wrap so that is it is stretched to a length corresponding to 30% of the resting thigh circumference during each revolution around the limb.

### Usability testing

#### User-based evaluation

The time to completion for task scenarios was 2.3 ± 1.2 min and the number of errors was 1 ± 1. In the order that scenarios were given to participants, time to task completion was 3.3 ± 1.4 min for the first scenario, 1.8 ± 1.2 min for the second scenario, and 1.8 ± 0.6 min for the third scenario. There was a total of 11 incidents among all participants during the completion of task scenarios. Incidents were categorized as navigation problems (4), data input problems (2), and difficulty interpreting recommendations (5). A summary of the type of incidents occurring during each task are presented in Table 4. When prompted to select equipment for implementing BFR, participants selected a manual thigh blood pressure cuff 80% of the time, knee wraps/elastic bands 10% of the time, and automated cuff systems 10% of the time. All participants selecting to use a manual thigh blood pressure cuff indicated that they did not have access to equipment for measuring AOP and utilized the application to estimate AOP. All task scenarios were completed “Successfully” and resulted in participants properly screening and determining an appropriate cuff pressure to utilize during exercise with BFR with all patient scenarios.

**TABLE 4:** Type and frequency of incidents occurring during user-based evaluations.

Step	Navigation	Data input	Interpreting recommendation
Step 1: Medical Screening	• Difficulty locating output from medical screening (2) • Unsure how to proceed to next step (2)	• Forgot to hit “Submit” button	NA
Step 2: Selecting Equipment	NA	NA	• Thought that width of cuff referred to limb circumference
Step 3: Determining Cuff Pressure	NA	• Entered units within input field and was given “error”	• Confusion about AOP value in output (2) • Difficulty remembering stratification level from medical screening • Problems interpreting elastic wrap directions

#### Perceived usability

Composite SUS scores were 94 ± 5, which corresponded to an “A+” on the curved graded scale and ranked within the 96–100th percentile range of industry SUS standards. Composite and individual item SUS responses are presented in Table 5. All individual item responses were above benchmarks for an SUS composite score of 80.

**TABLE 5:** System Usability Scale (SUS) responses and normative data.

Participant	Item number										Composite
	1	2	3	4	5	6	7	8	9	10	
1	5	1	5	1	5	1	5	1	5	1	100
2	4	1	5	1	5	2	5	1	4	1	92.5
3	5	1	4	1	4	1	5	2	5	1	92.5
4	5	1	5	1	4	1	5	1	5	1	97.5
5	4	1	4	1	4	1	5	2	4	1	87.5
Mean ± SD	4.6 ± 0.6	1 ± 0	4.6 ± 0.6	1 ± 0	4.4 ± 0.6	1.2 ± 0.5	5 ± 0	1.4 ± 0.6	4.6 ± 0.6	1 ± 0	94 ± 5 (A+)
Benchmark	≥3.4	≤2.4	≥3.7	≤1.9	≥3.6	≤2.2	≥3.7	≤2.3	≥3.7	≤2.1	68 (C)
Benchmark	≥3.8	≤1.9	≥4.2	≤1.5	≥4.0	≤1.8	≥4.2	≤1.7	≥4.3	≤1.6	80 (A-)

#### Semi-structured interviews

**FIGURE 7:**
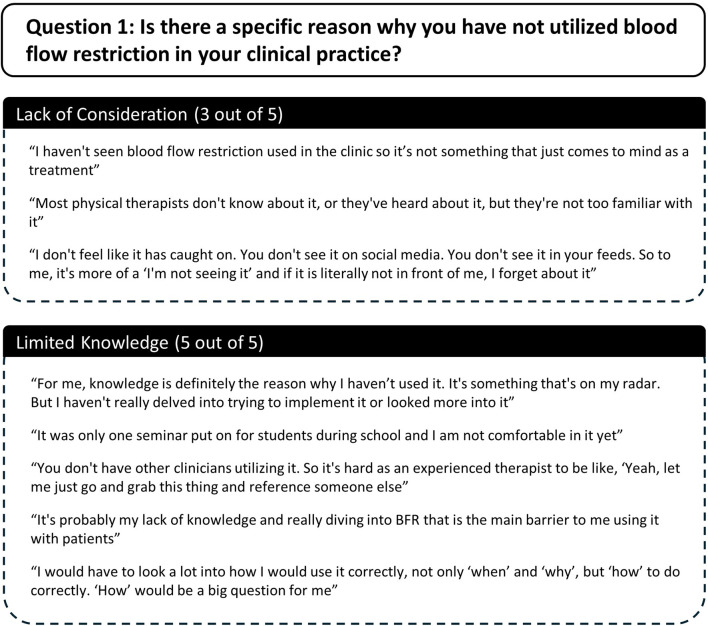
Participant responses to semi-structured interview question 1.

**FIGURE 8:**
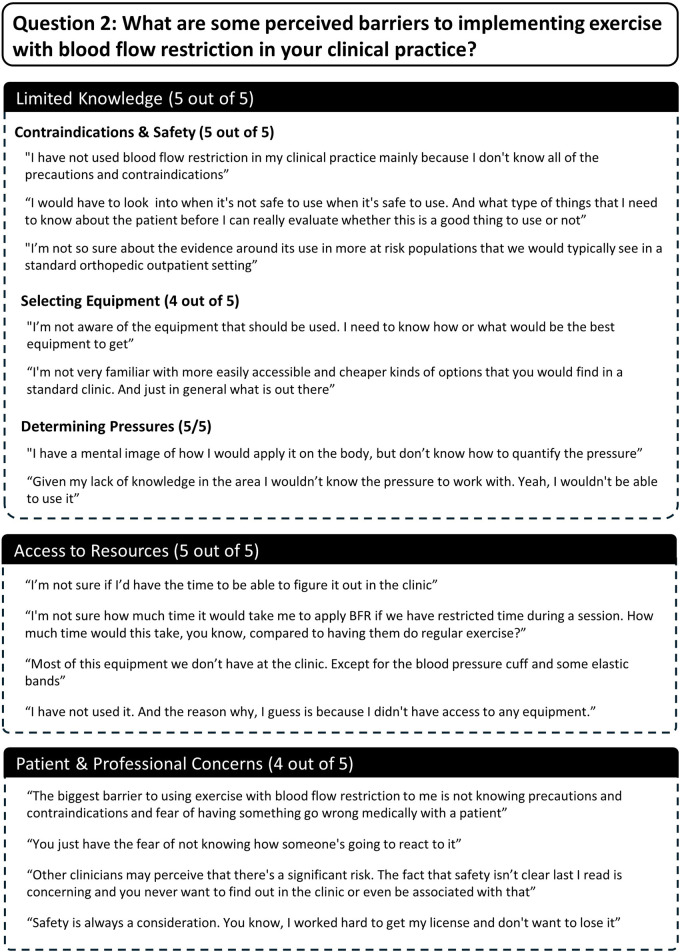
Participant responses to semi-structured interview question 2.

**FIGURE 9:**
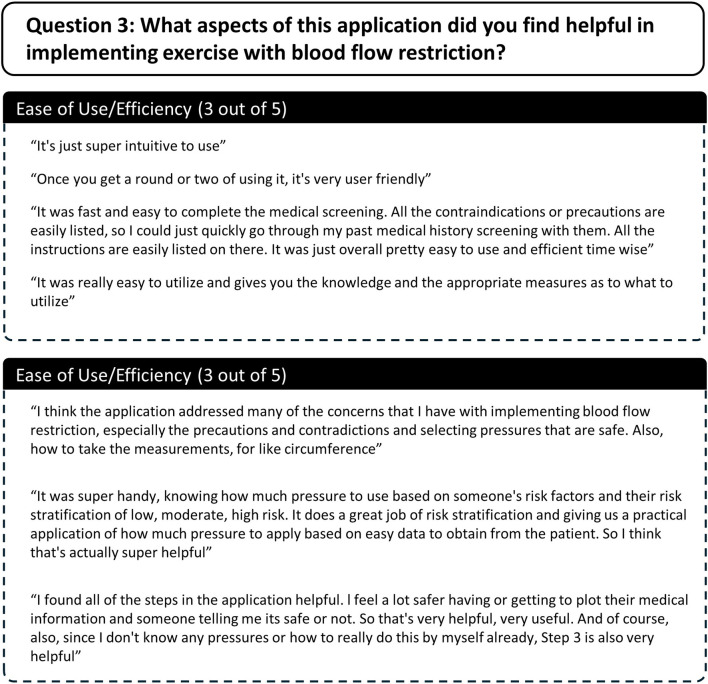
Participant responses to semi-structured interview question 3.

**FIGURE 10:**
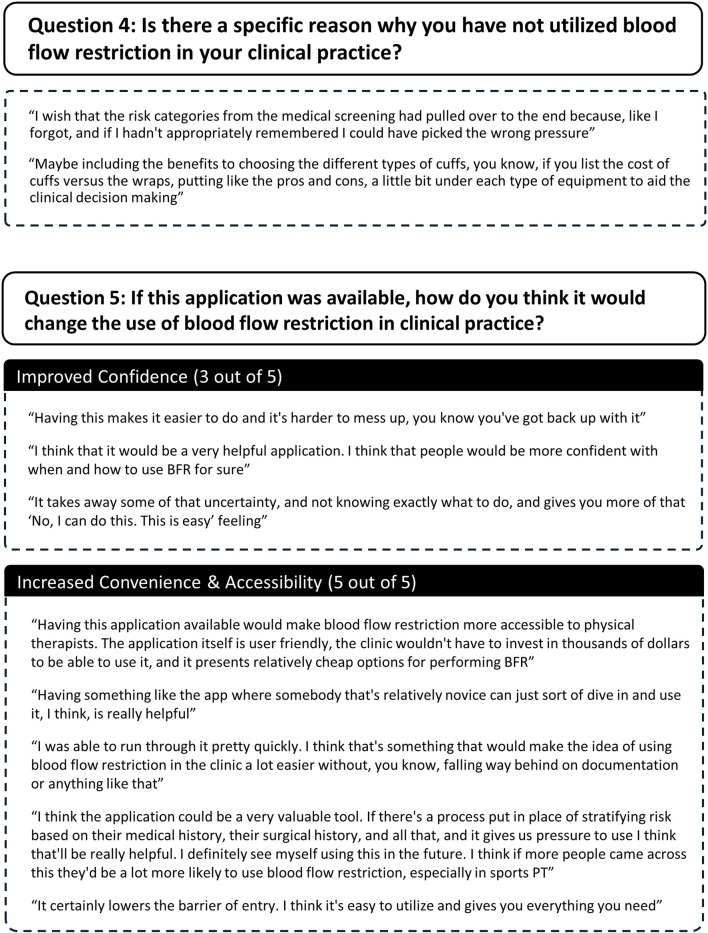
Participant responses to semi-structured interview question 4.

Several themes and subthemes emerged from qualitative analysis of participants semi-structured interview responses. Themes and subthemes from participants responses to each question are described here and in Figures 7–10.

Question 1 Is there a specific reason why you have not utilized blood flow restriction in your clinical practice?

Two themes emerged as to why participants had not utilized exercise with BFR in their clinical practice including 1) lack of consideration, and 2) limited knowledge (Figure 7). Three out of five participants indicated that exercise with BFR was simply not a method that they often considered when treating patients. Furthermore, whether they had considered BFR or not, all participants reported that a lack of knowledge about BFR was a reason why they had not utilized it. Additionally, four participants commented that their lack of consideration and/or knowledge was due to limited exposure to exercise with BFR. Two participants stated that they had not seen BFR used in the clinic by colleagues, one participant commented on limited exposure during their schooling, and another commented on limited exposure in the media.

Question 2 What are some perceived barriers to implementing exercise with blood flow restriction in your clinical practice?

Three themes emerged as barriers to using exercise with BFR and included 1) limited knowledge, 2) limited access to resources, and 3) patient and professional concerns (Figure 8). All participants reported that a lack of knowledge pertaining to the implementation of BFR presented a barrier to using it. Furthermore, several subthemes were identified related to specific areas of limited knowledge. These included uncertainty surrounding contraindications and safety of performing BFR (5/5 participants), what equipment to utilize for performing BFR (4/5 participants), and determining pressures to apply during exercise (5/5 participants). All participants also reported that limited access to resources posed a barrier. Four out of five participants mentioned having limited access to equipment for performing BFR and two out five commented on having limited time to implement BFR. Lastly, four out of five participants mentioned that the risk of BFR causing adverse events in patients and/or threatening their professional status were barriers to its use.

Question 3 What aspects of this application did you find helpful in implementing exercise with blood flow restriction?

Two themes were identified pertaining aspects of the application that participants found helpful including 1) ease of use/efficiency and 2) useful content and features (Figure 9). All participants agreed that the web-based application was easy to use and time efficient. Participants also agreed that the content and features included within each step of the application addressed gaps in knowledge and were useful for implementing exercise with BFR. All participants specifically mentioned Step 1: Medical screening and Step 2: Determining Restriction Pressure being particularly helpful.

Question 4 How could this application be improved to help you implement blood flow restriction more confidently?

Three participants provided feedback on how the application could be improved. Two participants did not give any suggestions. Suggestions included 1) better integrating the results of the medical screening into the selection of pressures to use during exercise with BFR and 2) including more information about the benefits and drawbacks of selecting certain types of equipment for implementing BFR (Figure 10).

Question 5 If this application was available, how do you think it would change the use of blood flow restriction in clinical practice?

Two themes emerged related to how the web-based application would change the use of exercise with BFR in clinical settings and included 1) improved confidence with using BFR and 2) increased accessibility of BFR (Figure 10). Three out of five participants reported that having the application would increase practitioners’ confidence of using exercise with BFR. All participants stated that the web-based application would make exercise with BFR more accessible to practitioners. Specifically, they reported that the web-based application lowered the requisite knowledge needed to implement exercise with BFR (3/5 participants), lowered costs associated with BFR use (1/5 participants), and would make implementing BFR more time efficient (1/5 participants). Furthermore, two participants commented that the web-based application would make practitioners more likely to utilize exercise with BFR.

## Discussion

### Main findings

The purpose of this study was to describe the development of a preliminary web-based application to aid in the implementation of exercise with BFR and to conduct preliminary usability testing in physical therapists to identify issues and provide feedback for further development. Our main findings were that 1) the web-based application can serve as an evidence-based decision support tool for implementation of exercise with BFR, 2) physical therapists found the functionality and content of the web-based application helpful for implementing exercise with BFR, and 3) usability of the web-based application was high in physical therapists possessing no previous experience using exercise with BFR. Lastly, several areas for improvement were identified including the addition of more informational content about BFR equipment, enhancing integration of steps and functions, and making user recommendations easier to interpret.

### Application development

To the best of our knowledge, we are the first group to report the development of a decision support tool for evidence-based implementation of exercise with BFR. We utilized a commercially available website builder and interactive Shiny applications to construct a preliminary web-based application. Functional steps included in the preliminary design were aimed at addressing methodological barriers to the implementation of BFR that have been previously identified (Rolnick et al., 2021) in practitioners. Specifically, we developed a medical screening tool as well as a decision-making pathway for equipment selection and pressure determination that were based on an aggregated synthesis of existing literature. In agreement with the findings of Scott et al. (2024), participants in this study identified several barriers to utilizing BFR. These barriers included limited knowledge and education, insufficient access to resources such as equipment and time, and safety concerns. Specifically, participants highlighted key knowledge gaps that hindered their use of BFR, including a lack of understanding of contraindications and safety precautions, uncertainty about how to select appropriate equipment for BFR, and confusion regarding how to determine appropriate pressure settings for restrictive devices. Our results suggest that the content and functions included within our web-based application were helpful in addressing each of these perceived barriers. Participants stated that having the web-based application would increase their confidence implementing exercise with BFR, lower the requisite knowledge required to use BFR, and would make practitioners more likely to utilize the modality in clinical practice. While our medical screening tool, decision-making process, and equipment recommendations are not validated and do not reflect expert consensus, our results provide proof of concept. Notably, these results suggest that an application incorporating a similarly designed screening process and decision-making pathway is usable by physical therapists and may help address key barriers to BFR implementation in clinical settings. These results also highlight the need for the development of a consensus-based medical screening tool, like that developed by the Austrian Institute of Sport (2021), and a clear, practical set of step-by-step guidelines for BFR implementation. Future research should aim to better define relevant risk factors to enable effective screening without being overly exclusionary, and to clarify the safety profiles of different BFR devices and how device selection should align with individual risk factors.

An interesting finding was that participants selected to utilize a thigh blood pressure cuff to implement exercise with BFR during most (80%) of the hypothetical task scenarios using the web-based application. Furthermore, all participants choosing to utilize this equipment indicated that they did not have access to handheld or ultrasound Doppler for directly measuring AOP. Thus, all participants determined exercising cuff pressures for this device by estimating AOP using our prediction equation (Wedig et al., 2024). Accordingly, the strategies selected for implementing BFR were limited and not representative of the most common methodologies currently used in clinical settings (Scott et al., 2024). These results may have been due to our study design and the participants’ prior knowledge. The hypothetical patient scenarios provided to participants focused specifically on implementing BFR for the lower-body with the goal of improving lower-body strength. This naturally directed participants toward lower-body cuff options, limiting exploration of other implementation pathways within the application. Additionally, all participants had no prior experience with BFR exercise and limited knowledge of how to implement it or what types of equipment were available. When asked to select from the recommended equipment, they were instructed to choose the option they were most likely to have access to and use in their clinical setting. As a result, many selected more familiar and readily available equipment such as manual blood pressure cuffs. These data do however indicate that a major strength of our web-based application was providing more accessible options for implementing BFR that did not require specialized equipment. Accordingly, the application may help to enhance equipment accessibility by introducing practitioners to more practical means of implementing BFR. Feedback about how to improve the content of the application was minimal. One participant suggested including more information about the various BFR equipment types would be helpful in making a more informed clinical decision when choosing which equipment to utilize with patients.

### Usability testing

Results indicated that our web-based application had a high degree of usability within our sample of physical therapists. Composite SUS scores ranked highly among industry standards, well above values suggested to represent “Excellent” usability (Bangor et al., 2009), and higher than scores previously reported for other applications being used by physical therapists (Baschung Pfister et al., 2020). Furthermore, all individual item responses were well above benchmarks for an acceptable SUS score. Importantly, these data indicated that the web-based application was effective, efficient, and satisfactory to use. Effectiveness of a system referrers to how well a systems performance meets the task that it was designed for. Compared to other technological systems utilized by physical therapists (Baschung Pfister et al., 2020; Rhodes et al., 2019), our application demonstrated a relatively high task completion rate. During user-based evaluation there was a 100% success rate in which all participants successfully implemented exercise with BFR in each of the hypothetical scenarios that they were presented with. This included successful medical screening of patients for BFR inclusion, selecting appropriate equipment for performing BFR, and selecting an appropriate cuff pressure to utilize based on risk stratification. Efficiency refers to how much time and effort are required to use a system to achieve a desired task. Using the web-based application, participants were able to complete all steps of implementing exercise with BFR in under 3 min. After becoming familiarized to the web-based application, time to completion decreased by almost half, suggesting that participants were able to quickly learn the system interface. Additionally, participants described the web-based application as being “easy to utilize,” “user friendly,” “intuitive,” and/or “time efficient” in their interview responses. Finally, satisfaction refers to how pleasant a system is to utilize and its ability to favor positive attitudes from a user. Interview responses largely suggested that participants experience using the web-based application was positive. Several participants stated that they would use this application if it was available.

No critical design problems in the web-based application were identified. Incidents occurring during user-based evaluations helped to identify minor issues related to navigation, data input, and interpreting recommendations provided by the application. Navigation problems largely occurred during the medical screening. Specifically, the layout of the medical screening Shiny application made it difficult for users to locate the risk stratification output. Additionally, after identifying the risk stratification level in the Shiny application, participants had difficulty navigating back to the top of the webpage to select the resulting risk level and move onto the next step. Collectively, feedback suggested that the results from medical screening were not well integrated into the other functions of the application. For example, when determining exercising cuff pressures, participants were given pressure recommendations for all risk stratification levels and some participants had difficulty remembering the assigned risk level provided during medical screening. One participant suggested that cuff pressure recommendations in Step 3 be provided only for the patient previously screened. This reflects a limitation of our overall application development (i.e., using a website with embedded Shiny applications). Shiny applications do not directly interface with the website, making it challenging to integrate results into future steps. Notably, several participants had difficulty interpreting the pressure to utilize based on the output from the AOP estimation Shiny application. Specifically, a patient’s AOP was listed in the output along with recommended exercising pressures and several participants were confused about what the AOP value represented. Lastly, one participant selecting to utilize elastic bands had difficulty interpreting how to apply the wraps based on the patient’s limb circumference. Accordingly, several recommendations to enhance usability of the web-based application include 1) re-designing the layout of the medical screening Shiny application so that the risk stratification output is easier to locate, 2) better integrating the results of medical screening into the determination of cuff pressure, 3) defining AOP and indicating more clearly the recommended pressures to use during exercise, and 4) improving instructions for setting tightness with elastic wraps. Collectively, development of a more integrated application may help to overcome many of the issues identified by users.

### Limitations

There are several noteworthy limitations to this study. First, participants were given a limited number of similar hypothetical task scenarios during user-based evaluation and thus did not experience all possible scenarios for implementing exercise with BFR within the web-based application. For example, all task scenarios focused on implementation of exercise with BFR in the lower body. Additionally, almost all participants chose to utilize the same equipment and methods for determining cuff pressure. Therefore, feedback related to alternative content and functions within the application was limited. These limitations were partly due to our use of a small number of participants with no prior experience implementing BFR, whose selection of methodologies and equipment was likely influenced by their limited knowledge of BFR practices. Second, use of the web-based application by practitioners was carried out virtually and with hypothetical scenarios where all patient information was easily provided. Thus, the generalizability of these results to use of the application in real world clinical practice is limited (Kjeldskov and Skov, 2007). Third, given the exploratory nature of this pilot study a single coder was used for thematic analysis which introduces a potential source of bias. Finally, our preliminary medical screening process and overall algorithm for BFR decision making require validation before they can be confidently recommended for real-world use. Collectivity, the results from this pilot study should be interpreted cautiously.

### Future directions

Future efforts will focus on three key areas of improvement for the application. First, we will enhance the user interface to improve overall usability. This includes transitioning from the current web-based prototype to a fully integrated standalone application, allowing for more seamless interaction between core features (e.g., medical screening, equipment selection, and pressure recommendations) which are currently constrained by the limitations of embedding Shiny applications within a website. Future iterations will also integrate user feedback, including redesigning the medical screening interface, clarifying AOP outputs and pressure recommendations, and refining guidance for elastic wrap application. Second, we aim to contribute to the development and validation of a consensus-based BFR screening tool and a set of implementation recommendations, similar to our proposed recommendations focused on equipment selection and cuff pressure determination. Our findings suggest that such resources could help address key barriers to BFR adoption among practitioners. Lastly, we will continue conducting usability testing as the application evolves. To enhance ecological validity and ensure a comprehensive evaluation across diverse use cases, future testing will include a broader range of hypothetical and real-world clinical scenarios. A larger, more diverse sample of practitioners with varying levels of BFR experience will be involved to reduce bias in implementation strategies and better reflect common clinical practices. It will also be important to evaluate whether the application’s algorithm unintentionally biases users toward certain strategies. In-person testing with practicing clinicians in real-world settings will be prioritized to assess practical usability. Additionally, concerns related to patient data confidentiality and the assumption of risk associated with BFR exercise will need to be thoroughly addressed before the application is ready for clinical use. These efforts aim to address current limitations of our preliminary application and support the development of a more robust, user-friendly, and clinically effective tool for implementing BFR exercise.

## Conclusion

Our preliminary web-based application presents a promising tool to help physical therapists implement safe and effective exercise with BFR. Through the application we were able to provide evidence-based guidance for medically screening potential BFR candidates, selecting appropriate equipment to utilize for performing BFR, and determining appropriate cuff pressures. The application’s core content and features appear to address many of the major barriers that physical therapists with limited experience in BFR face when attempting to incorporate it into clinical practice. Additionally, the application was effective and efficient in helping physical therapists to make appropriate decisions related to the implementation of exercise with BFR. Several areas for improvement were identified which will help to enhance the usability of this application. This work serves as an initial step in a broader research agenda aimed at constructing and validating a consensus-based screening tool, refining BFR implementation recommendations, and further developing a validated application for clinical use.

## Data Availability

The raw data supporting the conclusions of this article will be made available by the authors, without undue reservation.

## Abbreviations

BFR, blood flow restriction; SUS, system usability scale; AOP, arterial occlusion pressure.

## References

[B1] Abe T. Mouser J. G. Dankel S. J. Bell Z. W. Buckner S. L. Mattocks K. T. (2019). A method to standardize the blood flow restriction pressure by an elastic cuff. Scand. J. Med. Sci. Sports 29 (3), 329–335. 10.1111/sms.13340 30468528

[B2] Alsobhi M. Khan F. Chevidikunnan M. F. Basuodan R. Shawli L. Neamatallah Z. (2022). Physical therapists' knowledge and attitudes regarding artificial intelligence applications in health care and rehabilitation: cross-sectional study. J. Med. Internet Res. 24 (10), e39565. 10.2196/39565 36264614 PMC9634519

[B3] American Physical Therapy Association (2018). What to know about blood flow restriction. Available online at: https://www.apta.org/patient-care/interventions/blood-flow-restriction/what-to-know-about-blood-flow-restriction-training (Accessed March 17, 2023).

[B4] Aniceto R. R. Da Silva Leandro L. (2022). Practical blood flow restriction training: new methodological directions for practice and research. Sports Med. Open 8 (1), 87. 10.1186/s40798-022-00475-2 35763185 PMC9240154

[B5] Åström W. Sahlin W. (2022). User experience and acceptability of a mixed reality system for rehabilitation: from an occupational-and physical therapy perspective.

[B6] Austrailian Institute of Sport (2021). Blood flow restriction training guidelines. Available online at: https://www.ais.gov.au/position_statements/best_practice_content/blood-flow-restriction-training-guidelines (Accessed June 17, 2025).

[B7] Bangor A. Kortum P. Miller J. (2009). Determining what individual sus scores mean: adding an adjective rating scale. J. Usability Stud. 4 (3), 114–123.

[B8] Bangor A. Kortum P. T. Miller J. T. (2008). An empirical evaluation of the system usability scale. Int. J. Hum-Comput Int. 24 (6), 574–594. 10.1080/10447310802205776

[B9] Baschung Pfister P. Tobler-Ammann B. Knols R. H. De Bruin E. D. De Bie R. A. (2020). Usability and acceptance of an interactive tablet-based exercise application: a mixed methods study. Front. Digital Health 2, 578281. 10.3389/fdgth.2020.578281 PMC8521963 34713051

[B10] Bell Z. W. Dankel S. J. Spitz R. W. Chatakondi R. N. Abe T. Loenneke J. P. (2020). The perceived tightness scale does not provide reliable estimates of blood flow restriction pressure. J. Sport Rehabil. 29 (4), 516–518. 10.1123/jsr.2018-0439 31553951

[B11] Bennett H. Slattery F. (2019). Effects of blood flow restriction training on aerobic capacity and performance: a systematic review. J. Strength Cond. Res. 33 (2), 572–583. 10.1519/jsc.0000000000002963 30531417

[B12] Bevana N. Kirakowskib J. Maissela J. (1991) “What is usability,” in Proceedings of the 4th international conference on HCI, 1–6.

[B13] Boland D. M. Neufeld E. V. Ruddell J. Dolezal B. A. Cooper C. B. (2016). Inter-and intra-rater agreement of static posture analysis using a mobile application. J. Phys. Ther. Sci. 28 (12), 3398–3402. 10.1589/jpts.28.3398 28174460 PMC5276769

[B14] Boulos M. N. Wheeler S. Tavares C. Jones R. (2011). How smartphones are changing the face of mobile and participatory healthcare: an overview, with example from ecaalyx. Biomed. Eng. Online 10, 24. 10.1186/1475-925x-10-24 21466669 PMC3080339

[B15] Brandner C. R. May A. K. Clarkson M. J. Warmington S. A. (2018). Reported side-effects and safety considerations for the use of blood flow restriction during exercise in practice and research. Tech. Orthop. 33 (2), 114–121. 10.1097/bto.0000000000000259

[B16] Braun V. Clarke V. (2006). Using thematic analysis in psychology. Qual. Res. Psychol. 3 (2), 77–101. 10.1191/1478088706qp063oa

[B17] Brekke A. F. Sørensen A. N. Buhr C. Johannesdottír Í O. Jakobsen T. L. (2020). The validity and reliability of the handheld oximeter to determine limb occlusion pressure for blood flow restriction exercise in the lower extremity. Int. J. Sports Phys. Ther. 15 (5), 783–791. 10.26603/ijspt20200783 33110698 PMC7575150

[B18] Brooke J. (1996). Sus-a quick and dirty usability scale. Usability Eval. Industry 189 (194), 4–7.

[B19] Castle J. P. Tramer J. S. Turner E. H. Cotter D. Mcgee A. Abbas M. J. (2023). Survey of blood flow restriction therapy for rehabilitation in sports medicine patients. J. Orthop. 38, 47–52. 10.1016/j.jor.2023.03.007 36969302 PMC10030811

[B20] Cirilo-Sousa M. D. S. Lemos J. B. Poderoso R. Araújo R. C. T. D. Aniceto R. R. Pereira P. M. G. (2019). Predictive equation for blood flow restriction training. Rev Bras Med. do Esporte 25, 494–497. 10.1590/1517-869220192506186803

[B21] Clarkson M. J. May A. K. Warmington S. A. (2019). Chronic blood flow restriction exercise improves objective physical function: a systematic review. Front. Physiol. 10, 1058. 10.3389/fphys.2019.01058 31496953 PMC6712096

[B22] Cognetti D. J. Sheean A. J. Owens J. G. (2022). Blood flow restriction therapy and its use for rehabilitation and return to sport: physiology, application, and guidelines for implementation. Arthrosc. Sports Med. Rehabil. 4 (1), e71–e76. 10.1016/j.asmr.2021.09.025 35141538 PMC8811521

[B23] Colapietro M. Lee J. Vairo G. (2022). Use of blood flow restriction training between north american sports medicine practitioners: a questionnaire-based survey. Archives Phys. Med. Rehabilitation 103 (12), e143–e144. 10.1016/j.apmr.2022.08.816

[B24] Corrêa H. L. Neves R. V. P. Deus L. A. Maia B. C. H. Maya A. T. Tzanno-Martins C. (2021a). Low-load resistance training with blood flow restriction prevent renal function decline: the role of the redox balance, angiotensin 1-7 and vasopressin. Physiol. Behav. 230, 113295. 10.1016/j.physbeh.2020.113295 33340514

[B25] Corrêa H. L. Neves R. V. P. Deus L. A. Souza M. K. Haro A. S. Costa F. (2021b). Blood flow restriction training blunts chronic kidney disease progression in humans. Med. Sci. Sports Exerc 53 (2), 249–257. 10.1249/mss.0000000000002465 32826635

[B26] Cristina-Oliveira M. Meireles K. Spranger M. D. O'leary D. S. Roschel H. Peçanha T. (2020). Clinical safety of blood flow-restricted training? A comprehensive review of altered muscle metaboreflex in cardiovascular disease during ischemic exercise. Am. J. Physiol. Heart Circ. Physiol. 318 (1), H90–H109. 10.1152/ajpheart.00468.2019 31702969 PMC7002866

[B27] Cuffe M. Novak J. Saithna A. Strohmeyer H. S. Slaven E. (2022). Current trends in blood flow restriction. Front. Physiology 13, 882472. 10.3389/fphys.2022.882472 PMC9298746 35874549

[B28] Deutsch J. E. Romney W. Reynolds J. Manal T. J. (2015). Validity and usability of a professional association’s web-based knowledge translation portal: american physical therapy association’s ptnow.org. BMC Med. Inf. Decis. Mak. 15, 79–12. 10.1186/s12911-015-0178-y PMC4599310 26450415

[B29] Fahs C. A. Loenneke J. P. Rossow L. M. Tiebaud R. S. Bemben M. G. (2012). Methodological considerations for blood flow restricted resistance exercise. J. trainology 1 (1), 14–22. 10.17338/trainology.1.1_14

[B30] Fini E. M. Salimian M. Ahmadizad S. (2021). Responses of platelet cd markers and indices to resistance exercise with and without blood flow restriction in patients with type 2 diabetes. Clin. Hemorheol. Microcirc. 80 (3), 281–289. 10.3233/CH-211229 34511492

[B31] Formiga M. F. Fay R. Hutchinson S. Locandro N. Ceballos A. Lesh A. (2020). Effect of aerobic exercise training with and without blood flow restriction on aerobic capacity in healthy young adults: a systematic review with meta-analysis. Int. J. Sports Phys. Ther. 15 (2), 175–187. 10.26603/ijspt20200175 32269850 PMC7134358

[B32] Franko O. I. Tirrell T. F. (2012). Smartphone app use among medical providers in acgme training programs. J. Med. Syst. 36 (5), 3135–3139. 10.1007/s10916-011-9798-7 22052129

[B33] Freitas E. D. S. Karabulut M. Bemben M. G. (2021). The evolution of blood flow restricted exercise. Front. Physiol. 12, 747759. 10.3389/fphys.2021.747759 34925056 PMC8674694

[B34] Fukuda T. Yasuda T. Fukumura K. Iida H. Morita T. Sato Y. (2013). Low-intensity kaatsu resistance exercises using an elastic band enhance muscle activation in patients with cardiovascular diseases. Int. J. kaatsu train. Res. 9 (1), 1–5. 10.3806/ijktr.9.1

[B35] Genov A. (2005). Iterative usability testing as continuous feedback: a control systems perspective. J. Usability Stud. 1 (1), 18–27.

[B36] Grønfeldt B. M. Lindberg Nielsen J. Mieritz R. M. Lund H. Aagaard P. (2020). Effect of blood-flow restricted vs heavy-load strength training on muscle strength: systematic review and meta-analysis. Scand. J. Med. Sci. Sports 30 (5), 837–848. 10.1111/sms.13632 32031709

[B37] Hansen D. Dendale P. Coninx K. Vanhees L. Piepoli M. F. Niebauer J. (2017). The european association of preventive cardiology exercise prescription in everyday practice and rehabilitative training (expert) tool: a digital training and decision support system for optimized exercise prescription in cardiovascular disease. Concept, definitions and construction methodology. Eur. J. Prev. Cardiol. 24 (10), 1017–1031. 10.1177/2047487317702042 28420250

[B38] Hartstein A. J. Verkuyl M. Zimney K. Yockey J. Berg-Poppe P. (2022). Virtual reality instructional design in orthopedic physical therapy education: a mixed-methods usability test. Simul. & Gaming 53 (2), 111–134. 10.1177/10468781211073646

[B39] Hennink M. Kaiser B. N. (2022). Sample sizes for saturation in qualitative research: a systematic review of empirical tests. Soc. Sci. Med. 292, 114523. 10.1016/j.socscimed.2021.114523 34785096

[B40] Hughes L. Mcewen J. (2021). Investigation of clinically acceptable agreement between two methods of automatic measurement of limb occlusion pressure: a randomised trial. BMC Biomed. Eng. 3 (1), 8. 10.1186/s42490-021-00053-9 33964963 PMC8105974

[B41] Hughes L. Paton B. Rosenblatt B. Gissane C. Patterson S. D. (2017). Blood flow restriction training in clinical musculoskeletal rehabilitation: a systematic review and meta-analysis. Br. J. Sports Med. 51 (13), 1003–1011. 10.1136/bjsports-2016-097071 28259850

[B42] Hughes L. Rolnick N. Franz A. Owens J. Swain P. M. Centner C. (2025). Blood flow restriction: methods and apparatus still matter. Br. J. Sports. Med. 59 (9), 623–625. 10.1136/bjsports-2024-109365 39919806 PMC12171415

[B43] Hughes L. Rosenblatt B. Gissane C. Paton B. Patterson S. D. (2018). Interface pressure, perceptual, and mean arterial pressure responses to different blood flow restriction systems. Scand. J. Med. Sci. Sports 28 (7), 1757–1765. 10.1111/sms.13092 29630752

[B44] International Organization for Standardization (2018). Iso 9241-11:2018 ergonomics of human-system interaction. Part 11 Usability Defin. Concepts.

[B45] Ishizaka H. Uematsu A. Mizushima Y. Nozawa N. Katayanagi S. Matsumoto K. (2019). Blood flow restriction increases the neural activation of the knee extensors during very low-intensity leg extension exercise in cardiovascular patients: a pilot study. J. Clin. Med. 8 (8), 1252. 10.3390/jcm8081252 31430903 PMC6723568

[B46] Jacobs E. Rolnick N. Wezenbeek E. Stroobant L. Capelleman R. Arnout N. (2023). Investigating the autoregulation of applied blood flow restriction training pressures in healthy, physically active adults: an intervention study evaluating acute training responses and safety. Br. J. Sports Med. 57, 914–920. 10.1136/bjsports-2022-106069 36604156

[B47] Jaspers M. W. Steen T. Van Den Bos C. Geenen M. (2004). The think aloud method: a guide to user interface design. Int. J. Med. Inf. 73 (11-12), 781–795. 10.1016/j.ijmedinf.2004.08.003 15491929

[B48] Jessee M. B. Buckner S. L. Dankel S. J. Counts B. R. Abe T. Loenneke J. P. (2016). The influence of cuff width, sex, and race on arterial occlusion: implications for blood flow restriction research. Sports Med. 46 (6), 913–921. 10.1007/s40279-016-0473-5 26820301

[B49] Jessee M. B. Dankel S. J. Buckner S. L. Mouser J. G. Mattocks K. T. Loenneke J. P. (2017). The cardiovascular and perceptual response to very low load blood flow restricted exercise. Int. J. Sports Med. 38 (08), 597–603. 10.1055/s-0043-109555 28651256

[B50] Kacin A. Rosenblatt B. Žargi T. G. Biswas A. (2015). Safety considerations with blood flow restricted resistance training. Ann. Kinesiol. 6 (1), 3–26.

[B51] Kambič T. Novaković M. Tomažin K. Strojnik V. Jug B. (2019). Blood flow restriction resistance exercise improves muscle strength and hemodynamics, but not vascular function in coronary artery disease patients: a pilot randomized controlled trial. Front. Physiol. 10, 656. 10.3389/fphys.2019.00656 31244668 PMC6581774

[B52] Kjeldskov J. Skov M. B. (2007). Studying usability in sitro: simulating real world phenomena in controlled environments. Int. J. Human-Computer Interact. 22 (1-2), 7–36. 10.1207/s15327590ijhc2201-02_2

[B53] Kong J. Li Z. Zhu L. Li L. Chen S. (2022). Comparison of blood flow restriction training and conventional resistance training for the improvement of sarcopenia in the older adults: a systematic review and meta-analysis. Sports Med. Health Sci. 5, 269–276. 10.1016/j.smhs.2022.12.002 38314044 PMC10831374

[B54] Laprade R. F. Monson J. K. Schoenecker J. (2021). Editorial commentary: blood flow restriction therapy continues to prove effective. Arthroscopy 37 (9), 2870–2872. 10.1016/j.arthro.2021.04.073 34481627

[B55] Lewis J. R. Brown J. Mayes D. K. (2015). Psychometric evaluation of the emo and the sus in the context of a large-sample unmoderated usability study. Int. J. Hum-Comput Int. 31 (8), 545–553. 10.1080/10447318.2015.1064665

[B56] Lewis J. R. Sauro J. (2018). Item benchmarks for the system usability scale. J. Usability Stud. 13 (3).

[B57] Lima-Soares F. Pessoa K. A. Torres Cabido C. E. Lauver J. Cholewa J. Rossi F. (2022). Determining the arterial occlusion pressure for blood flow restriction: pulse oximeter as a new method compared with a handheld doppler. J. Strength Cond. Res. 36 (4), 1120–1124. 10.1519/jsc.0000000000003628 32379239

[B58] Lixandrão M. E. Ugrinowitsch C. Berton R. Vechin F. C. Conceição M. S. Damas F. (2018). Magnitude of muscle strength and mass adaptations between high-load resistance training versus low-load resistance training associated with blood-flow restriction: a systematic review and meta-analysis. Sports Med. 48 (2), 361–378. 10.1007/s40279-017-0795-y 29043659

[B59] Loenneke J. P. Allen K. M. Mouser J. G. Thiebaud R. S. Kim D. Abe T. (2015). Blood flow restriction in the upper and lower limbs is predicted by limb circumference and systolic blood pressure. Eur. J. Appl. Physiol. 115 (2), 397–405. 10.1007/s00421-014-3030-7 25338316

[B60] Loenneke J. P. Fahs C. A. Rossow L. M. Sherk V. D. Thiebaud R. S. Abe T. (2012a). Effects of cuff width on arterial occlusion: implications for blood flow restricted exercise. Eur. J. Appl. Physiol. 112 (8), 2903–2912. 10.1007/s00421-011-2266-8 22143843 PMC4133131

[B61] Loenneke J. P. Pujol T. J. (2009). The use of occlusion training to produce muscle hypertrophy. J. Strength Cond. 31 (3), 77–84. 10.1519/ssc.0b013e3181a5a352

[B62] Loenneke J. P. Wilson J. M. Marín P. J. Zourdos M. C. Bemben M. G. (2012b). Low intensity blood flow restriction training: a meta-analysis. Eur. J. Appl. Physiol. 112 (5), 1849–1859. 10.1007/s00421-011-2167-x 21922259

[B63] Loenneke J. P. Wilson J. M. Wilson G. J. Pujol T. J. Bemben M. G. (2011). Potential safety issues with blood flow restriction training. Scand. J. Med. Sci. Sports 21 (4), 510–518. 10.1111/j.1600-0838.2010.01290.x 21410544

[B64] Lu Y. Patel B. H. Kym C. Nwachukwu B. U. Beletksy A. Forsythe B. (2020). Perioperative blood flow restriction rehabilitation in patients undergoing acl reconstruction: a systematic review. Orthop. J. Sports Med. 8 (3), 2325967120906822. 10.1177/2325967120906822 32232065 PMC7097877

[B65] Madarame H. Kurano M. Fukumura K. Fukuda T. Nakajima T. (2013). Haemostatic and inflammatory responses to blood flow-restricted exercise in patients with ischaemic heart disease: a pilot study. Clin. Physiol. Funct. Imaging 33 (1), 11–17. 10.1111/j.1475-097X.2012.01158.x 23216760

[B66] Malekyian Fini E. Ahmadizad S. Salimian M. Motefakker M. Mokhtari Andani F. Fath Tabar L. (2021). The effect of two methods of resistance training with and without blood flow restriction on coagulation indices and blood glucose levels in type 2 diabetic patients. Med. J. Mashhad Univ. Med. Sci. 64 (3), 3060–3071. 10.22038/mjms.2021.18772

[B67] Maramba I. Chatterjee A. Newman C. (2019). Methods of usability testing in the development of ehealth applications: a scoping review. Int. J. Med. Inf. 126, 95–104. 10.1016/j.ijmedinf.2019.03.018 31029270

[B68] Mattocks K. T. Jessee M. B. Counts B. R. Buckner S. L. Mouser J. G. Dankel S. J. (2017). The effects of upper body exercise across different levels of blood flow restriction on arterial occlusion pressure and perceptual responses. Physiol. Behav. 171, 181–186. 10.1016/j.physbeh.2017.01.015 28088558

[B69] Mcewen J. A. Owens J. G. Jeyasurya J. (2019). Why is it crucial to use personalized occlusion pressures in blood flow restriction (bfr) rehabilitation? J. Med. Biol. Eng. 39 (2), 173–177. 10.1007/s40846-018-0397-7

[B70] Mills N. Elder M. Boyce M. Evdokas M. Ives S. (2021). The knowledge and use of blood flow restriction therapy in a sample of physical therapists in the United States. Res. Health Med. 1 (1). 10.53520/rdhs2021.10422

[B71] Muntaner-Mas A. Martinez-Nicolas A. Lavie C. J. Blair S. N. Ross R. Arena R. (2019). A systematic review of fitness apps and their potential clinical and sports utility for objective and remote assessment of cardiorespiratory fitness. Sports Med. 49 (4), 587–600. 10.1007/s40279-019-01084-y 30825094 PMC6422959

[B72] Nakajima T. Kurano M. Sakagami F. Iida H. Fukumura K. Fukuda T. (2010). Effects of low-intensity kaatsu resistance training on skeletal muscle size/strength and endurance capacity in patients with ischemic heart disease. Int. J. Kaatsu Train. Res. 6 (1), 1–7. 10.3806/ijktr.6.1

[B73] Nakajima T. Morita T. Sato Y. (2011). Key considerations when conducting kaatsu training. Int. J. kaatsu train. Res. 7 (1), 1–6. 10.3806/ijktr.7.1

[B74] Nascimento D. D. C. Rolnick N. Neto I. V. S. Severin R. Beal F. L. R. (2022). A useful blood flow restriction training risk stratification for exercise and rehabilitation. Front. Physiol. 13, 808622. 10.3389/fphys.2022.808622 35360229 PMC8963452

[B75] Nast I. Scheermesser M. Ernst M. Sommer B. Schmid P. Weisenhorn M. (2024). Usability of a visual feedback system to assess and improve movement disorders related to neck pain: perceptions of physical therapists and patients. Heliyon 10 (5), e26931. 10.1016/j.heliyon.2024.e26931 38434337 PMC10907800

[B76] Nielsen J. Landauer T. K. (1993) “A mathematical model of the finding of usability problems,” in Proceedings of the INTERACT′93 and CHI′93 conference on human factors in computing systems, 206–213.

[B77] Ogawa H. Nakajima T. Shibasaki I. Nasuno T. Kaneda H. Katayanagi S. (2021). Low-intensity resistance training with moderate blood flow restriction appears safe and increases skeletal muscle strength and size in cardiovascular surgery patients: a pilot study. J. Clin. Med. 10 (3), 547. 10.3390/jcm10030547 33540756 PMC7867301

[B78] Organisation I. S. (1999). Iso 13407: human-centred design processes for interactive systems. Geneva: ISO.

[B79] Patterson S. D. Brandner C. R. (2018). The role of blood flow restriction training for applied practitioners: a questionnaire-based survey. J. Sports Sci. 36 (2), 123–130. 10.1080/02640414.2017.1284341 28143359

[B80] Patterson S. D. Hughes L. Warmington S. Burr J. Scott B. R. Owens J. (2019). Blood flow restriction exercise: considerations of methodology, application, and safety. Front. Physiol. 10, 533. 10.3389/fphys.2019.00533 31156448 PMC6530612

[B81] Peart D. J. Balsalobre-Fernández C. Shaw M. P. (2019). Use of mobile applications to collect data in sport, health, and exercise science: a narrative review. J. Strength Cond. Res. 33 (4), 1167–1177. 10.1519/jsc.0000000000002344 29176384

[B82] Peres S. C. Pham T. Phillips R. (2013) “Validation of the system usability scale (sus) sus in the wild,” in Proceedings of the human factors and ergonomics society annual meeting. Los Angeles, CA: SAGE Publications Sage CA, 192–196.

[B83] Pitsillides A. Stasinopoulos D. Mamais I. (2021). Blood flow restriction training in patients with knee osteoarthritis: systematic review of randomized controlled trials. J. Bodyw. Mov. Ther. 27, 477–486. 10.1016/j.jbmt.2021.04.015 34391274

[B84] Rhodes J. K. Schindler D. Rao S. M. Venegas F. Bruzik E. T. Gabel W. (2019). Multiple sclerosis performance test: technical development and usability. Adv. Ther. 36, 1741–1755. 10.1007/s12325-019-00958-x 31054035 PMC6824297

[B85] Rolnick N. Kimbrell K. Cerqueira M. S. Weatherford B. Brandner C. (2021). Perceived barriers to blood flow restriction training. Front. Rehabil. Sci. 14. 10.3389/fresc.2021.697082 PMC9397924 36188864

[B86] Rolnick N. Kimbrell K. De Queiros V. (2023). Beneath the cuff: often overlooked and under-reported blood flow restriction device features and their potential impact on practice-a review of the current state of the research. Front. Physiol. 14, 1089065. 10.3389/fphys.2023.1089065 37064884 PMC10099250

[B87] Scott B. R. Marston K. J. Owens J. Rolnick N. Patterson S. D. (2024). Current implementation and barriers to using blood flow restriction training: insights from a survey of allied health practitioners. J. Strength & Cond. Res. 38 (3), 481–490. 10.1519/jsc.0000000000004656 38088873

[B88] Sieljacks P. Knudsen L. Wernbom M. Vissing K. (2018). Body position influences arterial occlusion pressure: implications for the standardization of pressure during blood flow restricted exercise. Eur. J. Appl. Physiol. 118 (2), 303–312. 10.1007/s00421-017-3770-2 29196847

[B89] Slysz J. Stultz J. Burr J. F. (2016). The efficacy of blood flow restricted exercise: a systematic review & meta-analysis. J. Sci. Med. Sport 19 (8), 669–675. 10.1016/j.jsams.2015.09.005 26463594

[B90] Spitz R. W. Wong V. Bell Z. W. Viana R. B. Chatakondi R. N. Abe T. (2022). Blood flow restricted exercise and discomfort: a review. J. Strength Cond. Res. 36 (3), 871–879. 10.1519/JSC.0000000000003525 32058360

[B91] Srikesavan C. S. Williamson E. Eldridge L. Heine P. Adams J. Cranston T. (2017). A web-based training resource for therapists to deliver an evidence-based exercise program for rheumatoid arthritis of the hand (isarah): design, development, and usability testing. J. Med. Internet Res. 19 (12), e411. 10.2196/jmir.8424 29237581 PMC5745347

[B92] Ventola C. L. (2014). Mobile devices and apps for health care professionals: uses and benefits. P t 39 (5), 356–364. 24883008 PMC4029126

[B93] Virzi R. A. (1990) “Streamlining the design process: running fewer subjects,” in Proceedings of the human factors society annual meeting. Los Angeles, CA: SAGE Publications Sage CA, 291–294.

[B94] Virzi R. A. (1992). Refining the test phase of usability evaluation: how many subjects is enough? Hum. Factors 34 (4), 457–468. 10.1177/001872089203400407

[B95] Wedig I. J. Lennox I. M. Petushek E. J. Mcdaniel J. Durocher J. J. Elmer S. J. (2024). Development of a prediction equation to estimate lower-limb arterial occlusion pressure with a thigh sphygmomanometer. Eur. J. Appl. Physiol. 124 (4), 1281–1295. 10.1007/s00421-023-05352-8 38001245

[B96] Wellmon R. H. Gulick D. T. Paterson M. L. Gulick C. N. (2016). Validity and reliability of 2 goniometric mobile apps: device, application, and examiner factors. J. Sport Rehabil. 25 (4), 371–379. 10.1123/jsr.2015-0041 27632853

[B97] Wong M. L. Formiga M. F. Owens J. Asken T. Cahalin L. P. (2018). Safety of blood flow restricted exercise in hypertension: a meta-analysis and systematic review with potential applications in orthopedic care. Tech. Orthop. 33 (2), 80–88. 10.1097/bto.0000000000000288

